# High Genetic Variation in the *COI* Gene Complicates Molecular Identification of *Eimeria* Species in Goats from South Korea

**DOI:** 10.3390/ani16193102

**Published:** 2026-10-02

**Authors:** Hyunjin Je, Daeun Kim, Rochelle A. Flores, Binh T. Nguyen, Aaron Paul R. Serdeña, Won Gi Yoo, Ho-Seong Cho, Wongi Min, Woo H. Kim

**Affiliations:** 1 Institution of Animal Medicine, College of Veterinary Medicine, Gyeongsang National University, Jinju 52828, Republic of Korea; hjssjd29@gmail.com (H.J.); daun1106@gnu.ac.kr (D.K.); floresrochellea@gmail.com (R.A.F.); thanhbinhcnty@gmail.com (B.T.N.); arserdena@gnu.ac.kr (A.P.R.S.); wongimin@gnu.ac.kr (W.M.); 2 Laboratory of Veterinary Parasitology, College of Veterinary Medicine, Seoul National University, Seoul 08826, Republic of Korea; wongi.yoo@snu.ac.kr; 3 Biosafety Research Institute, College of Veterinary Medicine, Jeonbuk National University, Iksan 54596, Republic of Korea; hscho@jbnu.ac.kr

**Keywords:** goat, coccidiosis, *Eimeria*, South Korea, cytochrome c oxidase subunit I (*COI*)

## Abstract

Despite the growth in South Korea’s goat industry, epidemiological data on goat coccidiosis—an intestinal disease caused by *Eimeria* spp., causing diarrhea and productivity losses, particularly in kids—remain limited. In 2025, fecal samples from 67 farms across 12 regions in Gyeongsangnam-do were analyzed for oocyst detection, oocysts per gram (OPG) quantification, and cytochrome c oxidase subunit I (*COI*) sequencing. Farm-level prevalence was high at 83.6% (56/67; range, 66.7–100%), with a mean OPG of 1136 (range: 250–7033) among positive samples. Morphologically, the observed oocysts resembled *E. christenseni*, *E. arloingi*, and *E*. *hirci*. *COI*-based BLAST analysis showed high identity with *E. christenseni* and *E. arloingi*, whereas phylogenetic analysis revealed affinities extending to *E. hirci* and sequences from cattle (*E. bovis*, *E. zuernii*) and sheep (*E. ahsata*). However, substantial *COI* heterogeneity, pooled sampling, and potential preferential amplification during Sanger sequencing limited confident species assignment. Here, findings demonstrate farm-level *Eimeria* detection but do not establish clinical or subclinical coccidiosis since clinical status was not systematically assessed. Nevertheless, these data establish a useful epidemiological and genetic baseline and highlight the need for multi-locus analysis (18S rRNA, internal transcribed spacer [ITS], and *COI*) to improve species-level resolution for surveillance and control strategies.

## Simple Summary

Coccidiosis is an intestinal parasitic disease caused by *Eimeria* spp. that manifests as diarrhea in affected animals. Although the goat industry in South Korea has expanded in response to growing consumer demand for goat products, systematic data on goat coccidiosis remain scarce. In this study, we assessed the farm-level prevalence and genetic diversity of *Eimeria* species in domestic goats by screening fecal samples from 67 farms across Gyeongsangnam-do in 2025. *Eimeria* oocysts were detected in more than 83% of the farms, and their morphological features closely resembled those of the pathogenic species *E. christenseni*, *E. arloingi*, and *E. hirci*. Sequencing of the mitochondrial cytochrome c oxidase subunit I gene (*COI*), a widely used molecular marker for apicomplexan parasites, revealed extensive sequence variation that blurred the genetic boundaries among goat-, cattle- and sheep-derived *Eimeria* strains, making definitive species-level identification difficult. Collectively, these findings show that *Eimeria* infection is widespread among goat farms in Gyeongsangnam-do and indicate that complementary genetic markets, including 18s rRNA and internal transcribed spacer (ITS), supported by validated reference sequences, are needed for reliable species-level identification in goat coccidiosis.

## 1. Introduction

Coccidiosis is a parasitic disease caused by protozoa of the genus *Eimeria*, family *Eimeriidae*, and phylum *Apicomplexa*. *Eimeria* protozoa have a single-host life cycle consisting of asexual and sexual reproductive stages within the host’s intestinal epithelial cells and a developmental stage in the external environment [1,2]. Infection occurs when a host ingests infective oocysts through contaminated feed or water, leading to the release of sporocysts and sporozoites that subsequently invade the intestinal epithelial cells [3,4,5,6]. These infections cause impaired growth, reduced productivity, and significant economic losses in the livestock industry, including poultry, cattle, sheep, and goats [3,4,7,8,9,10,11].

In goats, more than 26 *Eimeria* species have been identified worldwide, with *E. arloingi*, *E. christenseni*, and *E. ninakohlyakimovae* recognized as the primary pathogenic species [10,12,13,14]. Despite variation in dominant species across geographic settings, goat coccidiosis is widespread globally. A recent global meta-analysis reported an overall prevalence of 62.9% for goat coccidiosis, with national pooled prevalence rates ranging from 16.4% to 100% across 56 countries [12]. Primary field surveys across Asia, Australia, Africa, Europe, and South America report positivity rates of 30.0–100%, within-herd prevalence reaching up to 100%, and frequent mixed-species co-infections [14,15,16,17,18,19,20,21,22,23,24,25,26,27]. Young goats under 6 months of age are generally susceptible to infection, and fecal oocyst excretion generally peaks in kids between 2 and 4 months of age, while clinical coccidiosis is recorded 2–4 weeks post-weaning [3,12,19]. Goats raised under intensive and semi-intensive management practices are at higher risk for *Eimeria* infection, and subclinically infected adult goats can continuously shed oocysts as asymptomatic reservoirs, predisposing susceptible kids to infection [3,15,18,21,28]. Clinically, goat coccidiosis typically manifests as diarrhea, with persistent disease leading to dehydration, weight loss, growth retardation, reduced feed efficiency, and fatal outcomes in severe cases [5,10,29]. At necropsy, affected intestines may exhibit papillary lesions or polyps with mucosal thickening, necrosis, and epithelial desquamation, which compromise the intestinal barrier and absorptive functions [5,18,24,26,29,30,31,32,33].

Traditional diagnosis of goat coccidiosis relies on copromicroscopic flotation and oocyst morphometry [3,7,10,12]. However, this approach is constrained by the significant morphological overlap among *Eimeria* species and the subjectivity of the examiner. To overcome this limitation, PCR-based molecular assays targeting multi-locus genetic markers, such as 18S rRNA, internal transcribed spacer (ITS), and cytochrome c oxidase (*COI*), enable species identification via sequencing with high sensitivity and specificity [34,35,36,37]. While such molecular platforms are supported by well-curated genomic repositories and species-specific primers in poultry, public sequence databases for goat *Eimeria* remain remarkably underrepresented [34,36]. This genomic deficit severely hinders accurate species differentiation in mixed co-infections, risks misclassifying the pathogenicity of unrecorded goat lineages, and restricts the practical application of routine molecular diagnostics in goat farms.

Global production of fresh or chilled goat meat reached 7.74 million tonnes in 2024, with Asia accounting for the largest share (73.2%, 1994–2024) [38]. South Korea reflects this expansion, with its goat population increasing by 74% from 243,520 in 2010 to 423,430 in 2023, and Gyeongsangnam-do alone accounts for 20.9% (2140 farms) of farms nationwide [39,40]. Despite this growth in the goat industry, the epidemiology of goat coccidiosis in South Korea remains poorly resolved, with limited systematic prevalence estimates and a lack of information on circulating *Eimeria* species. Species-level molecular identification of goat *Eimeria* is further constrained by the limited availability of validated ITS reference sequences and *COI*, whose discriminatory performance remains insufficiently evaluated. Given the high global prevalence and economic importance of coccidiosis, this study determines farm-level prevalence in Gyeongsangnam-do and characterizes species based on mitochondrial *COI* sequences to establish a useful molecular epidemiological baseline for species-level surveillance, improve diagnosis, and inform targeted goat coccidiosis control in South Korea.

## 2. Materials and Methods

### 2.1. Fecal Sample Collection

In 2025, goat fecal samples were collected from 67 farms, designated GN1 to GN67, in 12 cities and counties within Gyeongsangnam-do that agreed to participate in this study. A single representative pooled sample (approximately 10–20 g) was obtained per farm by randomly gathering fresh fecal matter from multiple points within the housing area, which was then placed into 50 mL conical tubes, transferred to the laboratory at Gyeongsang National University, and stored in a refrigerator (4 °C) until flotation analysis or DNA extraction. This pooled sampling approach was utilized to assess overall farm-level presence rather than individual-level infection.

### 2.2. Fecal Flotation and Examination

A portion of each pooled fecal sample was transferred to a new 50 mL conical tube, mixed with distilled water at a 1:2 to 1:5 (*w*/*v*) ratio, and left for a few minutes to soften. The feces were then thoroughly crushed and homogenized, followed by filtration through a layer of gauze. For flotation analysis, 1 mL of the filtered suspension was mixed with 9 mL of saturated NaCl solution prepared by dissolving 358 g of NaCl in 1 L of distilled water, resulting in a final 1:10 dilution. The mixture was immediately loaded onto McMaster slides for microscopic examination and oocyst counting.

After standing for 5 min, microscopic observation was conducted starting from one of the four corners and proceeding in a zigzag pattern for oocyst counting. The oocysts counted from both chambers and the total number of oocysts per gram of feces (OPG) were calculated as previously described, with modification using the following formula: OPG = [(oocyst count in chamber 1 + oocyst count in chamber 2)/(2 × 0.15)] × total dilution factor, where total dilution factor is the dilution of the sample with distilled water × dilution with saturated NaCl [41]. A sample was considered positive when at least one morphologically compatible *Eimeria* oocyst was observed in either McMaster chamber.

Qualitative morphological characterization of goat *Eimeria* oocysts was performed based on the criteria previously described in [21,42]. Representative oocysts from each *Eimeria* spp. positive farm samples (approximately or at least 50 oocysts per sample) were examined and photographed at 400× magnification using an Olympus IX70 Inverted Phase Contrast DIC Fluorescence Microscope (Olympus, Tokyo, Japan). Oocyst length and width were measured, while shape and the presence or absence of polar cap and micropyle were recorded.

### 2.3. DNA Extraction

The previously filtered suspension was centrifuged at 3130× *g* for 10 min to pellet the oocysts. After discarding the supernatant, saturated NaCl solution was added, and mixed thoroughly, and the mixture was centrifuged again at 3130× *g* for 10 min. The supernatant was recovered, and the pellet was washed with PBS at 1760× *g* for 5 min. This washing process was repeated 2–3 times. The final pellet was resuspended in 200–300 μL of PBS, and genomic DNA was extracted by bead beating using the QIAamp PowerFecal Pro Kit (QIAGEN, Hilden, Germany) according to the manufacturer’s instructions.

### 2.4. Polymerase Chain Reaction (PCR)

Only the positive samples verified by microscopic analysis were used for PCR. PCR amplification of the *COI* gene of *Eimeria* was conducted using the primers listed in Table 1 ([32,34]). PCR was carried out using Taq polymerase PCR PreMix following the manufacturer’s protocol (Bioneer, Daejeon, Republic of Korea) with forward and reverse primer stocks prepared at 10 pmol/μL, and PCR conditions were optimized following previously described protocols [34,43]. Briefly, the thermocycling conditions included an initial denaturation at 95 °C for 5 min, followed by 35 cycles of denaturation at 94 °C for 30 s, annealing at 55 °C for 30 s, and extension at 72 °C for 45 s. This was followed by a final extension step at 72 °C for 10 min. In addition, to prevent cross-contamination, filter tips were used throughout the sample preparation and PCR setup. A no-template control was also included in each PCR run.

The amplified PCR products were confirmed via 1% agarose gel electrophoresis in Tris-acetate-EDTA (TAE) buffer and purified using a QIAquick Gel extraction kit (QIAGEN, Hilden, Germany) following the manufacturer’s instructions. Bidirectional Sanger sequencing using the same primers as those used for PCR amplification was outsourced to Macrogen Inc. (Seoul, Republic of Korea). The sequence chromatograms were visually inspected, and low-quality bases were trimmed using Geneious Prime (version 2023.2, Biomatters Ltd., Auckland, New Zealand). Trimmed forward and reverse reads were subsequently assembled to generate a reliable consensus sequence for each sample. Samples displaying ambiguous or overlapping chromatogram peaks were resequenced to resolve uncertain base calls and generate high-confidence consensus sequences. To determine genetic relationships, sequence identities between the amplified gene sequences and existing *Eimeria* sp. *COI* sequences in GenBank (Available online: https://www.ncbi.nlm.nih.gov/genbank/ Available online: https://blast.ncbi.nlm.nih.gov/Blast.cgi, accessed on 19 June 2026).

To evaluate phylogenetic relationships among *Eimeria* species from ruminants (including *Eimeria christenseni* and *E. arloingi*) and chickens, multiple sequence alignment of the 56 *COI* sequences obtained in this study and 12 *COI* reference sequences retrieved from GenBank was performed using the MUSCLE algorithm integrated into the MEGA X software (v10.2.6). Both ends of the aligned sequences were manually trimmed to ensure equal sequence length. The best-fit nucleotide substitution model was evaluated using the Find Best DNA/Protein Models tool in MEGA X. Based on the lowest Bayesian Information Criterion (BIC) scores, a phylogenetic tree was constructed using the Maximum Likelihood (ML) method under the Hasegawa–Kishino–Yano model with gamma-distributed rate variation among sites (HKY+G). Gaps and missing data were handled using the partial deletion option with a site coverage cutoff of 95% in MEGA X. Furthermore, the reliability of the tree topology was assessed via bootstrap analysis with 1000 replicates.

### 2.5. In Silico Analysis of Goat Eimeria Genetic Sequences

In silico analysis was conducted on the available gene sequences retrieved from the NCBI GenBank database to evaluate target sequence conservation for PCR-based detection of goat coccidiosis. The search terms included “Goat *Eimeria*”, “*Eimeria christenseni*”, “*Eimeria arloingi*”, “*Eimeria hirci*”, and “*Eimeria ninakohlyakimovae*”. Sequences originated from goats were included regardless of gene region or sequence length (both full-length and partial sequences); however, sequences identified only at the genus level (i.e., *Eimeria* spp.) were excluded to ensure species-specific accuracy. Multiple sequence alignments were generated using Clustal Omega (v1.2.4) with default parameters, incorporating k-tuple matching and Profile HMM algorithms. Pairwise sequence identity percentages obtained directly from the Clustal Omega alignment output were used to evaluate sequence conservation and genetic variability among the analyzed sequences.

## 3. Results

### 3.1. Farm-Level Prevalence of Eimeria Infection

*Eimeria* oocysts were detected in the pooled fecal samples from 56 of 67 farms (farm-level prevalence, 83.6%), with regional positivity ranging from 66.7% to 100.0%. These results indicate that *Eimeria* infection was widely distributed across all surveyed regions. Among the positive farms, the mean pooled sample OPG was 1136. The pooled OPG values were ≤100 in 16 farms (28.6%), ≤1000 in 28 farms (50%), ≤5000 in 9 farms (16.1%), and >5000 in 3 farms (5.4%). Among regions represented by at least four farms, observed positivity was 100% in Hamyang; 88.9% in Jinju and Sacheon; 85.7% in Hadong, Goseong, and Geochang; and 66.7% in Geoje. Across all sampled regions, mean pooled-sample OPG ranged from 250 in Hapcheon to 7033 in Tongyeong (Table 2).

Morphological analysis revealed both unsporulated and sporulated oocysts. Following the comparative criteria of de Macedo et al., the morphometric assessment of goat *Eimeria* oocysts, based on the form, polar cap, micropyle, oocyst diameter, and sporocyst diameter, showed that several oocysts from the farm samples in this study morphologically resembled *E. christenseni*, *E. arloingi*, and *E. hirci* [21]. Examined oocysts possessed both a micropyle and a polar cap; however, due to the significant overlap and subtle differences among these morphotypes, definitive species identification could not be achieved, and no species-specific proportions were calculated. Representative photos of oocysts found in the farm samples that tested positive for *Eimeria* spp. are presented in Supplementary Figure S1.

### 3.2. Sequencing Analysis

**Figure 1:**
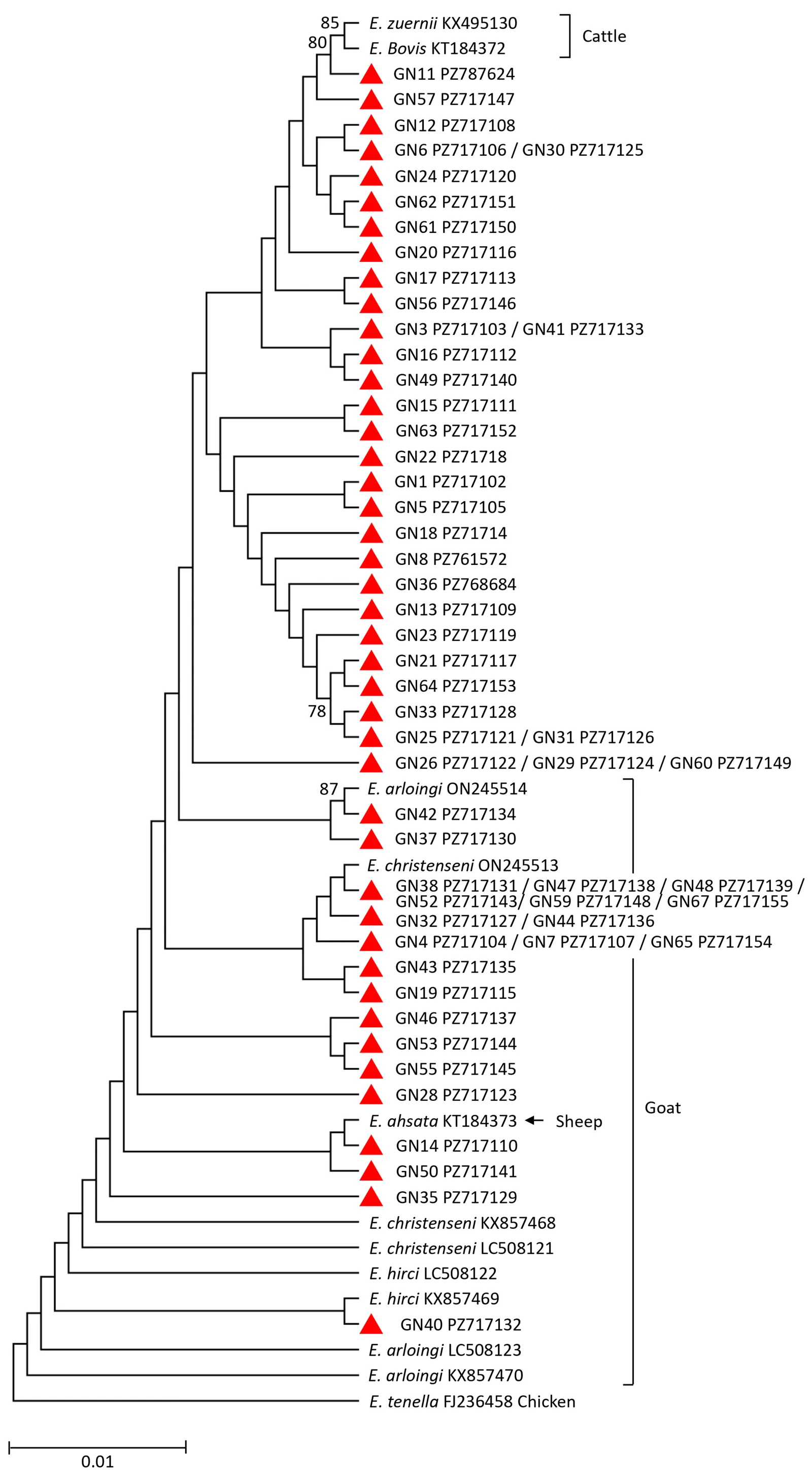
Phylogenetic tree constructed using the Maximum Likelihood method based on the partial *COI* sequences (522 bp) obtained in this study and representative sequences of other *Eimeria* species retrieved from GenBank. The best-fit model (HKY+G) was selected based on the lowest BIC score in MEGA X with 1000 bootstrap replicates. Sequences obtained in this study are indicated by ▲ with their respective GenBank accession numbers. Reference sequences retrieved from GenBank are presented with their respective species names and GenBank accession numbers. *Eimeria tenella* (FJ236458) was used as an outgroup. Numbers at the branches indicate bootstrap node support values ≥ 70%; values below 70% are omitted for clarity.

In the present study, 56 *Eimeria COI* sequences were obtained, with sequence lengths of approximately 522 bp. BLAST and phylogenetic analyses were performed using these sequences alongside reference *COI* sequences from *E. arloingi*, *E. christenseni*, other ruminant-derived *Eimeria* species (*E. bovis*, *E. zuernii*, and *E. ahsata*), and one avian outgroup (*E. tenella*) (Figure 1).

The BLAST analysis revealed that all sequences exhibited the highest sequence similarity to goat-derived species (91.57–99.81%), including *E. christenseni* (ON245513) and *E. arloingi* (ON245514) (Supplementary Table S1). In contrast, the phylogenetic analysis demonstrated more nuanced evolutionary relationships. A total of 13 samples (GN4, GN7, GN19, GN32, GN38, GN43-44, GN47-48, GN52, GN59, GN65, and GN67) were closely related to *E. christenseni* (ON245513); two samples (GN37 and GN42) clustered with *E. arloingi* (ON245514); and one sample (GN40) clustered with *E. hirci* (KX857469). Interestingly, despite being of goat origin, 15 samples (GN3, GN6, GN11, GN12, GN16-17, GN20, GN24, GN30, GN41, GN49, GN56-57, GN61, and GN62) positioned adjacent to the cattle-derived species, *E. bovis* (KT184372) and *E. zuernii* (KX495130). Additionally, 15 samples (GN1, GN5, GN8, GN13, GN15, GN18, GN21-23, GN25, GN31, GN33, GN36, GN63, and GN64) occupied an intermediate position between the cattle-derived clade and the *E. arloingi* clade, showing close genetic affinity to both lineages. Lastly, two samples (GN14 and GN50) were closely positioned with the sheep species *Eimeria ahsata* (KT184373), while eight samples (GN26, GN28, GN29, GN35, GN46, GN53, GN55, and GN60) formed an independent sub-cluster along with distinct single branches (Figure 1).

### 3.3. In Silico Analysis

The genetic targets selected for *Eimeria* sequence analysis were 18S rRNA, *COI*, and ITS (ITS1 and ITS2). Sequence availability in the GenBank database is restricted to four species—*E. christenseni*, *E. arloingi*, *E. hirci*, and *E. ninakohlyakimovae*—with no data currently available for other species (Table 3).

A comparison of the species’ 18S rRNA (90.5–99.9%) and *COI* (93.8–99.3%) sequences demonstrated a high similarity. In contrast, sequence similarity was lower for ITS1 (63.6–75.9%) and ITS2 (87.1%) (Table 4).

## 4. Discussion

### 4.1. Farm-Level Prevalence of Goat Eimeria Infection in Gyeongsangnam-do

In this study, we investigated *Eimeria* infection in Gyeongsangnam-do and revealed a farm-level prevalence of 83.6%, with *Eimeria* oocysts detected in 56 of 67 farms across 12 cities and counties. The high prevalence observed in this study concurs with previous farm-level prevalence previously reported in Australia (97%), Portugal (100%), and Greece (61%) [20,27,44]. Although no additional clinical investigation was conducted, the goats in Gyeongsangnam-do Province were found to shed oocysts that morphologically resemble both pathogenic and non-pathogenic *Eimeria* species. Although regional positivity ranged from 66.7% to 100%, unequal sampling among regions (1–16 farms) and the use of a pooled fecal sampling design limit direct comparison of prevalence and OPG values across regions. Nevertheless, the results here establish widespread *Eimeria* occurrence in Gyeongsangnam-do goat farms and underscore the importance of limiting environmental oocyst contamination, particularly in farms housing susceptible goats.

### 4.2. Morphological Identification of Eimeria Species

Morphological identification of *Eimeria* species is traditionally based on the characteristics of sporulated oocysts, including size, shape, and the presence or absence of specific elements such as a micropyle and a polar cap [21]. More than 26 species have been identified in goats, with *Eimeria arloingi*, *E. christenseni*, *E. hirci*, *E. ninakohlyakimovae*, *E. alijevi*, *E. caprina*, *E. caprovina*, *E. aspheronica*, and *E. jolchijevi* being the most frequently reported [12,21]. Among these nine species, *E. arloingi*, *E. christenseni*, and *E. hirci* are considered the most pathogenically significant and morphologically well-characterized; these three major species all possess both a micropyle and a polar cap, with their morphotypes ranging from ovoid to ellipsoid [45]. Their reported average dimensions are approximately 28 × 19 μm (*E. arloingi*), 38 × 24 μm (*E. christenseni*), and 23 × 18 μm (*E. hirci*), with *E. christenseni* being the largest and *E. hirci* the smallest [21]. Based on these morphometric criteria, oocysts observed in the fecal samples morphologically resemble those of *E. christenseni*, *E. arloingi*, and *E. hirci*. Previous reports based on microscopic examination have also reported high prevalence of these *Eimeria* species in goats in other Asian countries [14,19,28,46,47,48]. However, considerable variability in size, shape, and clarity of the micropyle and polar cap precludes definitive species identification in the current work. These variations and structural overlaps clearly illustrate the inherent limitations of microscopic analysis and concur with previous report [32].

### 4.3. Molecular (COI-Based) Identification and Its Limitations

Here, BLAST and phylogenetic analyses revealed a clear discrepancy between simple sequence similarity scores and actual phylogenetic relationships. BLAST similarity merely reflects the degree of nucleotide sequence identity to deposited GenBank references, rather than serving as a definitive criterion for objective and accurate species identification [49,50]. This is because the BLAST algorithm calculates local identity matches in conserved regions without accounting for phylogenetic evolutionary divergence [51].

Furthermore, the absence of clear detection of the highly pathogenic species *E. ninakohlyakimovae* highlights diagnostic limitations. Under controlled sporulation conditions, the morphological differentiation of *E. ninakohlyakimovae* may be possible, but this species exhibits substantial morphological overlap with other goat *Eimeria* species, thereby limiting reliable species-level identification by microscopy [21,52]. Moreover, pooling fecal material from multiple locations within each farm likely captured oocysts at heterogeneous stages of sporulation, which, together with frequent mixed-species infection, further constrained robust morphology-based species identification. In addition, *COI* reference sequences for *E. ninakohlyakimovae*, as of writing, are not available in GenBank. Although molecular identification using 18S rDNA has been reported, such studies remain scarce, and the availability of validated reference sequences is still limited [48,53]. Additionally, because direct sequencing preferentially recovers abundant templates, low-abundance co-infecting species such as *E. ninakohlyakimovae* may go undetected even when present [54]. Even if DNA from *E. ninakohlyakimovae* were available, the lack of validated reference sequences prevented molecular confirmation. Although recognized as one of the most prevalent goat *Eimeria* species, *E. ninakohlyakimovae* was not detected in the present study, consistent with previous reports in which it likewise remained undetected despite the use of 18S rDNA and *COI* barcoding techniques [14,15,32]. Thus, morphological overlap, the absence of a database for the target sequences, and sequencing constraints collectively restricted definitive identification of this pathogenic species.

Recent meta-analyses have shown that only about 47% of goat coccidiosis studies identified *Eimeria* parasites at the species level, and merely 4% utilized molecular biological methods [12]. Currently, no species-specific primers for goat *Eimeria* have been developed, and most molecular diagnoses rely on sequencing of the 18S rRNA or *COI* regions [15,55]. While the 18S rRNA gene is useful for amplification due to its high conservation, it has limited resolving power for distinguishing host specificity [46]. Consequently, the mitochondrial *COI* gene was targeted in this study as a primary marker due to its higher evolutionary rate and potentially greater discriminatory resolution [48].

In the comparison of intraspecific similarity among GenBank reference sequences, the *COI* sequences of chicken-derived *E. tenella* remained highly conserved, with a minimum identity of 98.28% (Supplementary Table S2). In contrast, the reference sequences for the three goat-derived species (*E. christenseni*, *E. arloingi*, and *E. hirci*) showed relatively lower intraspecific identities (93.56–97.49%). Notably, the sequence identities among the goat-derived sequences obtained in this study—93.82% for *E. christenseni* and 90.42% for *E. arloingi*—tended to be comparable to, or considerably lower than, those of the reference sequences described above, indicating a trend toward greater sequence variability. Several farm-derived sequences also formed separate subclades or basal branches, and goat sequences were not consistently resolved from sheep- or cattle-derived references, differing from the *COI*-based separation reported by Liang et al. [46]. These patterns demonstrate substantial *COI* heterogeneity and limited discriminatory power in the present dataset. However, since species identities were not independently validated, this heterogeneity cannot be interpreted as confirmed intraspecific variation or evidence of novel lineages.

Reference uncertainty further complicated phylogenetic interpretation. Of 101 GenBank records retrieved across 18S rRNA, ITS1, ITS2, and *COI* for goat *Eimeria*, nearly all were partial; only *E. christenseni* and *E. arloingi* possess sequences covering all four gene targets; and no validated *COI* reference was available for *E. ninakohlyakimovae* at the time of database retrieval. Moreover, the only sheep-derived *COI* sequence assigned to *E. ahsata* was reported with 18S rRNA data but lacked detailed morphological validation [55], limiting its value for comparison. This contrasts with chicken *Eimeria*, for which all seven recognized species are represented across the target loci, and ITS1 has been successfully applied to species differentiation [56,57]. However, marker performance in avian species cannot be extrapolated to goat *Eimeria*. A previous Korean goat study from Eumseong-gun, Chungcheongbuk-do similarly reported a sequence cluster distinct from available references [32]. Collectively, these observations highlight incomplete reference coverage and limited *COI* resolution that complicates species-level identification. However, the current available data are insufficient to determine whether this variation reflects previously uncharacterized lineages. Further, pooled sampling, potential mixed infections, and preferential amplification during Sanger sequencing limited species resolution. Reliable identification will require expanded multi-locus reference datasets derived from morphologically validated, preferably single-oocyst samples.

### 4.4. Epidemiological and Practical Significance

To date, systematic evidence on goat coccidiosis remains limited in South Korea. A previous survey (2009–2011) reported 67.6% prevalence among Korean native goats, while a clinical case from Eumseong-gun documented > 70,000 OPG and intestinal nodular lesions in affected kids [16,32]. The present detection of *Eimeria* on 83.6% of surveyed farms, therefore, establishes a useful baseline and demonstrates widespread farm-level occurrence of *Eimeria* in Gyeongsangnam-do. This finding is consistent with the broad global distribution of goat *Eimeria* and frequent reports of mixed infections involving pathogenic species [17,19,26,28,58,59]. However, the present study did not assess clinical disease, productivity, or economic losses and therefore cannot be inferred from farm-level positivity. Although uncertain species assignment limits differentiation between pathogenic and less pathogenic *Eimeria*, it does not preclude general clinical management. Intervention decisions should integrate age, clinical signs, individual OPG measurement, herd history, and, where available, pathological findings. Particular attention should be directed toward symptomatic young goats, and measures that reduce fecal contamination and environmental oocyst accumulation should be implemented.

### 4.5. Study Limitations and Future Directions

This study included a relatively small, geographically confined, and unevenly distributed sample of farms, limiting generalizability. Because each farm was represented by one pooled fecal sample and the ages of contributing animals were unavailable, positivity and OPG reflect farm-level detection and pooled shedding rather than individual- or age-specific prevalence and infection intensity. The use of McMaster method may also underestimate low-intensity infections, while the absence of systematic clinical assessment precluded classification as clinical or subclinical coccidiosis [13,60]. Species resolution is further constrained by overlapping morphologies, partial *COI* sequences, incomplete reference coverage, and potential mixed infections. Direct Sanger sequencing without cloning or single-oocyst separation may preferentially recover dominant templates and mask less abundant genotypes [61]. Consequently, the observed *COI* heterogeneity cannot be classified confidently as intraspecific variation, interspecific, or previously uncharacterized lineages. These taxonomic findings cannot be extrapolated directly to other livestock, and similarity to sheep or cattle sequences does not establish cross-host transmission. Instead, the broader implication is methodological. The partial *COI* sequencing of pooled samples may inadequately resolve mixed *Eimeria* infections, highlighting the need for host-validated reference databases and multi-locus or single-oocyst sequencing.

## 5. Conclusions

This study investigated the farm-level prevalence of *Eimeria* infection across 67 farms in 12 cities and counties within Gyeongsangnam-do, South Korea, in 2025. The analysis revealed a high farm-level prevalence, with 83.6% (56/67) of the examined farms, suggesting that *Eimeria* infection persists in Korean goat farms in Gyeongsangnam-do and can cause insidious economic losses. Neither morphometry nor partial *COI* sequencing enabled definitive species identification due to morphological overlap, potential mixed infections, preferential amplification, limited phylogenetic resolution, and incomplete reference coverage. Despite these constraints, the study established a useful epidemiological and genetic baseline, demonstrating both widespread farm-level occurrence and the diagnostic consequences of substantial *COI* sequence heterogeneity. Moreover, these findings expose a broader diagnostic gap in goat *Eimeria* and underscore the need for age-resolved surveillance and morphologically validated multi-locus reference datasets to enable reliable species-level identification, clarify distribution of pathogenic species, and guide risk-based coccidiosis control.

## Figures and Tables

**Table 1:** Primers for *COI* gene.

Gene	Primer	Sequence (5′ → 3′)	Size (bp)	Reference
*COI*	Forward	GTTTGGTTCAGGTGTTGGTTG	Up to 522	[34]
	Reverse	GTCATCATATGRTGTGCCCA		[32]

**Table 2:** Farm-level prevalence of *Eimeria* infection in Gyeongsangnam-do.

Location	No. of Tested Farms	No. of Negative Farms (%)	No. of Positive Farms (%)					OPG
			1–100	101–1000	1001–5000	>5000	Total	Mean
Hadong	7	1 (14.3)	1 (14.3)	4 (57.1)	1 (14.3)	0 (0)	6 (85.7)	600
Sancheong	16	5 (31.3)	6 (37.5)	4 (25)	1 (6.3)	0 (0)	11 (68.8)	300
Geoje	3	1 (33.3)	1 (33.3)	0 (0)	1 (33.3)	0 (0)	2 (66.7)	1267
Goseong	7	1 (14.3)	1 (14.3)	3 (42.9)	2 (28.6)	0 (0)	6 (85.7)	667
Sacheon	9	2 (11.1)	3 (33.3)	4 (44.4)	1 (11.1)	0 (0)	8 (88.9)	554
Jinju	9	2 (11.1)	2 (22.2)	4 (44.4)	1 (11.1)	1 (11.1)	8 (88.9)	3288
Changnyeong	1 *	0 (0)	0 (0)	1 (100)	0 (0)	0 (0)	1 (100)	967
Hamyang	4	0 (0)	1 (25)	1 (25)	2 (50)	0 (0)	4 (100)	800
Hapcheon	2 *	0 (0)	0 (0)	2 (100)	0 (0)	0 (0)	2 (100)	250
Namhae	1 *	0 (0)	0 (0)	1 (100)	0 (0)	0 (0)	1 (100)	300
Geochang	7	1 (14.3)	1 (14.3)	4 (57.1)	0 (0)	1 (14.3)	6 (85.7)	1245
Tongyeong	1 *	0 (0)	0 (0)	0 (0)	0 (0)	1 (100)	1 (100)	7033
Total	67	11 (16.4)	16 (23.9)	28 (41.8)	9 (13.4)	3 (4.5)	56 (83.6)	1136

* Data from regions with a small number of farms (*n* ≤ 2) are considered preliminary and should be interpreted with caution due to sample size constraints.

**Table 3:** Number of goat *Eimeria* sequences available in GenBank.

Target Gene	*E. christenseni*	*E. arloingi*	*E. hirci*	*E. ninakohlyakimovae*
18S rRNA	38	27	5	3
*COI*	6	10	5	0
ITS1	1	4	0	0
ITS2	1	1	0	0
Total	46	42	10	3

**Table 4:** Homology of target genes across goat *Eimeria* species available in GenBank.

Gene	*Eimeria* spp.	Min. (%)	Max (%)	No.
18S rRNA	*E. christenseni*, *E. arloingi*, *E. hirci*, *E. ninakohlyakimovae*	90.5	99.9	73
*COI*	*E. christenseni*, * E. arloingi*, *E. hirci*	93.8	99.3	21
ITS1	*E. christenseni*, *E. arloingi*	63.6	75.9	5
ITS2	*E. christenseni*, *E. arloingi*	87.1	87.1	2

## Data Availability

All sequences were deposited in GenBank (GenBank accession no. PZ717102–PZ717141, PZ717143–PZ717155, PZ761572, PZ768684, and PZ787624). The data presented in this study are available on request from the corresponding author. The data are not publicly available due to privacy and ethical restrictions.

## Supplementary Materials

The following supporting information can be downloaded at https://www.mdpi.com/article/10.3390/ani16193102/s1, Figure S1: Representative pictures of *Eimeria* spp. oocysts from goat fecal samples in this study.; Table S1: Percentage similarity of farm sample *COI* gene sequences to *Eimeria* spp.; Table S2: *COI* gene sequences identity of *Eimeria tenella* isolates available in Genbank.

## References

[1] López-Osorio S., Chaparro-Gutiérrez J.J., Gómez-Osorio L.M. (2020). Overview of poultry Eimeria life cycle and host-parasite interactions. Front. Vet. Sci..

[2] Nguyen B.T., Flores R.A., Kim T., Min W. (2026). Understanding Eimeria infection for the treatment and prevention of chicken coccidian parasites. Front. Cell. Infect. Microbiol..

[3] Chartier C., Paraud C. (2012). Coccidiosis due to *Eimeria* in sheep and goats, a review. Small Rumin. Res..

[4] Foreyt W.J. (1990). Coccidiosis and cryptosporidiosis in sheep and goats. Vet. Clin. N. Am. Food Anim. Pract..

[5] Khodakaram Tafti A., Mansourian M. (2008). Pathologic lesions of naturally occurring coccidiosis in sheep and goats. Comp. Clin. Pathol..

[6] Vercruysse J. (1982). The coccidia of sheep and goats in Senegal. Vet. Parasitol..

[7] Bangoura B., Bardsley K.D. (2020). Ruminant coccidiosis. Vet. Clin. Food Anim. Pract..

[8] Andrews A. (2013). Some aspects of coccidiosis in sheep and goats. Small Rumin. Res..

[9] Blake D.P., Knox J., Dehaeck B., Huntington B., Rathinam T., Ravipati V., Ayoade S., Gilbert W., Adebambo A.O., Jatau I.D. (2020). Re-calculating the cost of coccidiosis in chickens. Vet. Res..

[10] Bawm S., Htun L.L. (2021). Management and control of Eimeria infection in goats. Goat Science-Environment, Health and Economy.

[11] Vilatuña E., Cantón G., Ovelar M., Morrell E., Lloberas M., García J. (2025). Bovine coccidiosis: Retrospective study in Central Argentina. Vet. Parasitol. Reg. Stud. Rep..

[12] Ali E.A., Ghafar A., Angeles-Hernandez J.C., Yaseen M., Gauci C.G., Beveridge I., Baxendell S., Jabbar A. (2025). Global prevalence of *Eimeria* species in goats: A systematic review and meta-analysis. Front. Vet. Sci..

[13] Diao N.-C., Zhao B., Chen Y., Wang Q., Chen Z.-Y., Yang Y., Sun Y.-H., Shi J.-F., Li J.-M., Shi K. (2022). Prevalence of Eimeria spp. among goats in China: A systematic review and meta-analysis. Front. Cell. Infect. Microbiol..

[14] Dewi D.A., Nugraheni Y.R., Awaludin A., Ninditya V.I., Priyowidodo D., Nurcahyo R.W., Ekawasti F., Prastowo J. (2025). First molecular detection of *Eimeria* spp. in domestic goats from Java Island, Indonesia. Open Vet. J..

[15] Al-Habsi K., Yang R., Ryan U., Miller D.W., Jacobson C. (2017). Morphological and molecular characterization of three Eimeria species from captured rangeland goats in Western Australia. Vet. Parasitol. Reg. Stud. Rep..

[16] Gebeyehu E., Seo M., Jung B., Byun J., Oem J., Kim H., Kwak D. (2013). Prevalence of gastrointestinal parasites in Korean native goats (*Capra hircus aegagrus*). J. Anim. Plant Sci..

[17] Abo-Shehada M.N., Abo-Farieha H.A. (2003). Prevalence of Eimeria species among goats in northern Jordan. Small Rumin. Res..

[18] Silva L.M.R.d., Vila-Viçosa M.J.M., Nunes T., Taubert A., Hermosilla C., Cortes H.C.E. (2014). *Eimeria* infections in goats in Southern Portugal. Rev. Bras. Parasitol. Vet..

[19] Bawm S., Win T.Z.B., Win S.Y., Htun L.L., Nakao R., Katakura K. (2020). First detection of *Eimeria* species in Myanmar domestic goats with both microscopic and molecular methods. Parasite.

[20] Ali E.A., Ghafar A., Baxendell S., Gauci C., Beveridge I., Stevenson M.A., Jabbar A. (2026). Epidemiological insights into the prevalence of, and risk factors for, Eimeria species in Australian dairy goats. Vet. J..

[21] de Macedo L.O., Santos M.A.B., da Silva N.M.M., do Rêgo Barros G.M.M., Alves L.C., Giannelli A., Ramos R.A.N., de Carvalho G.A. (2019). Morphological and epidemiological data on Eimeria species infecting small ruminants in Brazil. Small Rumin. Res..

[22] Etsay K., Megbey S., Yohannes H. (2020). Prevalence of sheep and goat coccidiosis in different districts of Tigray region, Ethiopia. Niger. J. Anim. Sci..

[23] Elshafie E.I., Al-Habsi K., Ali H., ElTahir Y., Al-Kharousi K., Al-Hamrashdi A., Khojali M., Al-Batrani A.M., Johnson E.H. (2025). Gastrointestinal parasites and molecular characterization of Eimeria spp. among imported small ruminants in the Sultanate of Oman. Vet. Parasitol. Reg. Stud. Rep..

[24] Mohamaden W.I., Sallam N.H., Abouelhassan E.M. (2018). Prevalence of Eimeria species among sheep and goats in Suez Governorate, Egypt. Int. J. Vet. Sci. Med..

[25] Mpofu T.J., Nephawe K.A., Mtileni B. (2020). Prevalence of gastrointestinal parasites in communal goats from different agro-ecological zones of South Africa. Vet. World.

[26] Kheirandish R., Nourollahi-Fard S.R., Yadegari Z. (2014). Prevalence and pathology of coccidiosis in goats in southeastern Iran. J. Parasit. Dis..

[27] Silva L., Carrau T., Vila-Viçosa M., Musella V., Rinaldi L., Failing K., Cortes H., Taubert A., Hermosilla C. (2020). Analysis of potential risk factors of caprine coccidiosis. Vet. Parasitol. Reg. Stud. Rep..

[28] Faizal A., Rajapakse R. (2001). Prevalence of coccidia and gastrointestinal nematode infections in cross bred goats in the dry areas of Sri Lanka. Small Rumin. Res..

[29] Koudela B., Boková A. (1998). Coccidiosis in goats in the Czech Republic. Vet. Parasitol..

[30] Priti V., Jignesh P., Jatin P., Jeetendra R., Yash P., Thakor K., Panchal P. (2017). *Eimeria* species infection in surti goat kids. Int. J. Sci. Environ. Technol..

[31] Jyothi Chatta Y.C., Srinivasa Naik H., Yuvaraj C., Hruday N., Narmada S. (2024). Nodular lesions in the intestine of goat with coccidiosis: A case report. Int. J. Vet. Sci. Anim. Husb..

[32] Roh S.G., Kim J., Ku B.-K., Lee K. (2023). Case study: Pathological and phylogenetic analysis of coccidiosis in two goats with heavy infection of unrecorded *Eimeria* sp.. Parasitol. Int..

[33] Jolhe D., Poornima G., Ghosh R., Kumar G.D., Chandrahas S., Shubhangi A., Deepak P. (2019). Pathomorphological study of coccidiosis in goats (*Capra hircus*). Rumin. Sci..

[34] Schwarz R.S., Jenkins M.C., Klopp S., Miska K.B. (2009). Genomic analysis of *Eimeria* spp. populations in relation to performance levels of broiler chicken farms in Arkansas and North Carolina. J. Parasitol..

[35] Kawahara F., Zhang G., Mingala C.N., Tamura Y., Koiwa M., Onuma M., Nunoya T. (2010). Genetic analysis and development of species-specific PCR assays based on ITS-1 region of rRNA in bovine Eimeria parasites. Vet. Parasitol..

[36] Alam M.Z., Dey A.R., Parvin S., Akter S., Rony S.A. (2021). ITS1-PCR based identification of chicken *Eimeria* species in poultry litter from Mymensingh district, Bangladesh. J. Adv. Vet. Anim. Res..

[37] Ekawasti F., Wardhana A.H., Nepho F., Purwanto E.S., Sawitri D.H., Martindah E. Molecular Detection of Eimeria Zuernii in Cattle in Malang, East Java, Indonesia by Nested-PCR. Proceedings of the 1st International Conference for Health Research–BRIN (ICHR 2022).

[38] Food and Agriculture Organization of the United Nations FAOSTAT: Crops and Livestock Products—Production/Yield Quantities of Meat of Goat, Fresh or Chilled, World (Total). https://www.fao.org/faostat/en/#data/QCL.

[39] Lee Y.-G., Choi J.-Y. (2025). Analysis of Trends and Challenges in the Goat Industry [염소산업 동향 분석과 과제].

[40] Ministry of Agriculture, Food and Rural Affairs (2025). 2024 Statistics on Other Livestock [2024년 기타가축통계].

[41] Zhang R., Li H., Wang X., Jike R., Lu S., Hao L. (2025). Comparative analysis of pathogenicity and anticoccidial resistance of field-isolated Eimeria strains in Sichuan Province, China. Front. Vet. Sci..

[42] Soe A., Pomroy W. (1992). New species of *Eimeria* (Apicomplexa: Eimeriidae) from the domesticated goat *Capra hircus* in New Zealand. Syst. Parasitol..

[43] Yang R., Brice B., Bennett M.D., Eliott A., Ryan U. (2013). Novel *Eimeria* sp. isolated from a King’s skink (*Egernia kingii*) in Western Australia. Exp. Parasitol..

[44] Lianou D.T., Arsenopoulos K.V., Michael C.K., Papadopoulos E., Fthenakis G.C. (2022). Protozoan parasites in adult dairy small ruminants and potential predictors for their presence in faecal samples. Microorganisms.

[45] Levine N.D. (1985). Veterinary Protozoology.

[46] Liang G., Yang X., Liu D., Li Y., Wang J., Chen X., Zhao G., Song J. (2022). Molecular Characterization of 18S rDNA, ITS-1, ITS-2, and COI from *Eimeria christenseni* and *E. arloingi* in Goats from Shaanxi Province, Northwestern China. Animals.

[47] Yusof A.M., Isa M.L.M. (2016). Prevalence of gastrointestinal nematodiasis and coccidiosis in goats from three selected farms in Terengganu, Malaysia. Asian Pac. J. Trop. Biomed..

[48] Verma R., Sharma D.K., Gururaj K., Paul S., Banerjee P., Tiwari J. (2017). Molecular epidemiology and point mutations in ITS1 and 18S rDNA genes of *Eimeria ninakohlyakimovae* and *E. christenseni* isolated from Indian goats. Vet. Parasitol. Reg. Stud. Rep..

[49] Koski L.B., Golding G.B. (2001). The closest BLAST hit is often not the nearest neighbor. J. Mol. Evol..

[50] Cantarel B.L., Morrison H.G., Pearson W. (2006). Exploring the relationship between sequence similarity and accurate phylogenetic trees. Mol. Biol. Evol..

[51] Altschul S.F., Gish W., Miller W., Myers E.W., Lipman D.J. (1990). Basic local alignment search tool. J. Mol. Biol..

[52] Cavalcante A.C.R., Teixeira M., Monteiro J.P., Lopes C.W.G. (2012). *Eimeria* species in dairy goats in Brazil. Vet. Parasitol..

[53] Yamaguchi K., Matsubayashi M., Nishii J., Takenaka Y., Iwamoto C., Fujii K., Kojima K., Shibahara T. (2026). Hepatic coccidiosis caused by Eimeria ninakohlyakimovae in a goat (*Capra hircus*) in Japan. J. Vet. Med. Sci..

[54] Himsworth C.G., Byers K.A., Fernando C., Speerin L., Lee M.J., Hill J.E. (2020). When the sum of the parts tells you more than the whole: The advantage of using metagenomics to characterize *Bartonella* spp. infections in Norway rats (*Rattus norvegicus*) and their fleas. Front. Vet. Sci..

[55] Ogedengbe M.E., El-Sherry S., Ogedengbe J.D., Chapman H.D., Barta J.R. (2018). Phylogenies based on combined mitochondrial and nuclear sequences conflict with morphologically defined genera in the eimeriid coccidia (*Apicomplexa*). Int. J. Parasitol..

[56] You M.-J. (2014). Comparison and analysis of phylogenetic relationship based on internal transcribed spacer 1, ITS-1 fragment of *Eimeria tenella* and *Eimeria acervulina* from broiler chicken in Korea. Eurasian J. Vet. Sci..

[57] Flores R.A., Nguyen B.T., Cammayo P.L.T., Võ T.C., Naw H., Kim S., Kim W.H., Na B.-K., Min W. (2022). Epidemiological investigation and drug resistance of *Eimeria* species in Korean chicken farms. BMC Vet. Res..

[58] Al-Amery M., Hasso S. (2002). Laboratory diagnosis of novel species of *Theileria hirci*, *Eimeria caprovina* and *Eimeria pallida* in goats in Iraq. Small Rumin. Res..

[59] Wang C., Xiao J., Chen A., Chen J., Wang Y., Gao J., Zhu X. (2010). Prevalence of coccidial infection in sheep and goats in northeastern China. Vet. Parasitol..

[60] Maurizio A., Marchiori E., Tessarin C., Cassini R. (2023). Comparing pooled and individual samples for estimation of gastrointestinal strongyles burden and treatment efficacy in small ruminants. Vet. Parasitol..

[61] Ferreira S.C., Jarquín-Díaz V.H., Heitlinger E. (2023). Amplicon sequencing allows differential quantification of closely related parasite species: An example from rodent Coccidia (*Eimeria*). Parasites Vectors.

