# Supplementary material for: High Genetic Variation in the COI Gene Complicates Molecular Identification of Eimeria Species in Goats from South Korea
Source: Animals (Basel). 2026 Oct 2;16(19):3102. doi: 10.3390/ani16193102 (PMC13663317; doi:10.3390/ani16193102)
Supplement: Supplementary file 1 [file animals-16-03102-s001.zip › Supplementary materials.pdf]

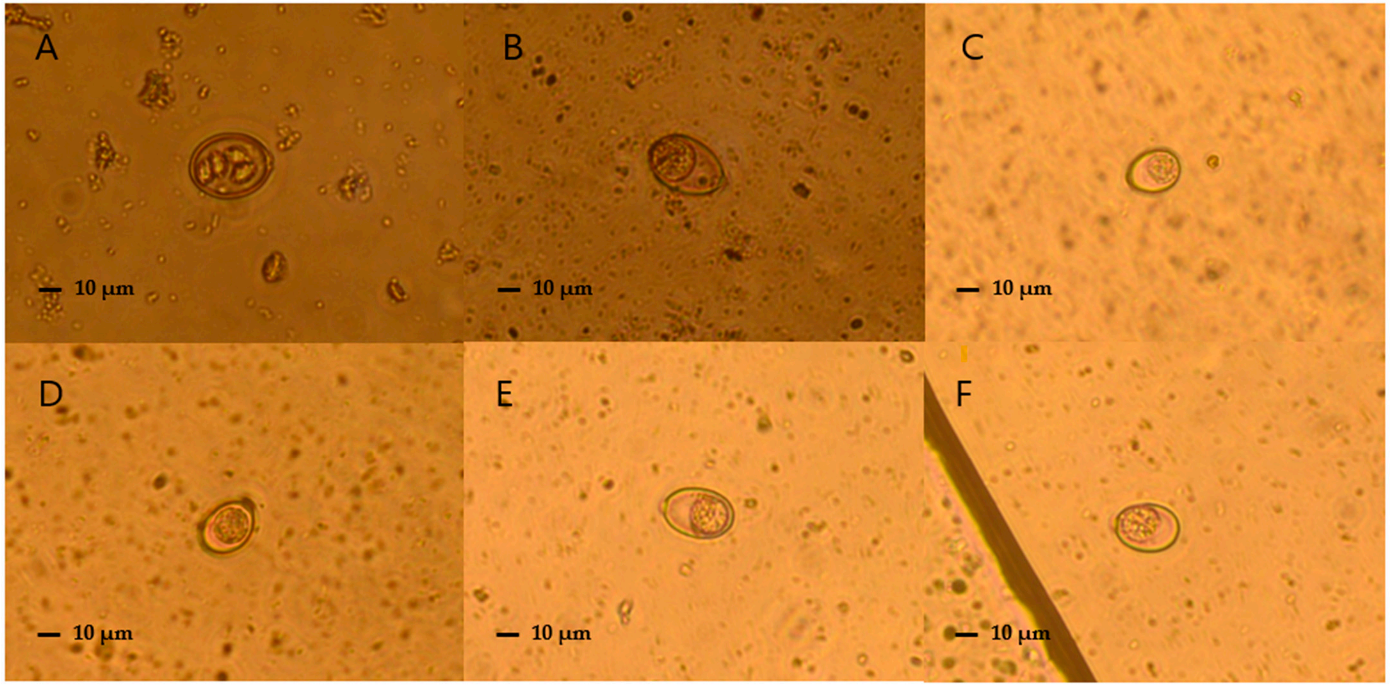

Supplementary Figure S1. Representative pictures of *Eimeria* spp. oocysts from goat fecal samples in this study. (A) and (B) *E. christenseni*; (C) and (D) *E. hirci*; (E) and (F) *E. arloingi*. (A) Sporulated oocyst sample from Jinju. (B–F) Unsporulated oocyst samples from different regions: (B, C, F) Geoje, (D) Geochang, and (E) Tongyeong. Oocysts in (A) and (B) were ovoid, measuring 36.23 × 25.12 μm and 35.74 × 23.67 μm, respectively; (C) and (D) were sub-spherical, measuring 22.22 × 18.84 μm and 23.67 × 19.32 μm, respectively; and (E) and (F) were ellipsoid, measuring 29.00 × 21.26 μm and 27.05 × 19.32 μm, respectively. Based on these morphological features,(A) and (B) morphologically resembled *E. christenseni*-like, (C) and (D) to *E. hirci*-like, and (E) and (F) to *E. arloingi*-like oocysts. Notably, all observed oocysts possessed both a micropyle and a polar cap, a common structural feature across the three morphotypes. *Eimeria spp.* oocysts were observed under light microscopy (×400). Scale bar = 10 μm

Supplementary Table S1. Percentage similarity of farm sample COI gene sequences to *Eimeria* spp.

Sample		Highest BLAST 1			Highest BLAST 2			Highest BLAST 3		
ID	Accession number	ID	Accession number	Percentage Similarity	ID	Accession number	Percentage Similarity	ID	Accession number	Percentage Similarity
GN1	PZ717102	*E. christenseni*	ON245513	98.47	*E. arloingi*	ON245514	98.08	*E. ashata*	KT184373	98.98
GN3	PZ717103	*E. arloingi*	ON245514	98.47	*E. christenseni*	ON245513	98.08	*E. ashata*	KT184373	96.18
GN4	PZ717104	*E. christenseni*	ON245513	99.43	*E. arloingi*	ON245514	98.08	*E. ashata*	KT184373	96.59
GN5	PZ717105	*E. arloingi*	ON245514	98.66	*E. christenseni*	ON245513	98.47	*E. ashata*	KT184373	96.59
GN6	PZ717106	*E. christenseni*	ON245513	99.23	*E. arloingi*	ON245514	98.85	*E. ashata*	KT184373	9639
GN7	PZ717107	*E. christenseni*	ON245513	99.43	*E. arloingi*	ON245514	98.08	*E. ashata*	KT184373	96.59
GN8	PZ761572	*E. christenseni*	ON245513	98.47	*E. arloingi*	ON245514	98.47	*E. ashata*	KT184373	96.59
GN11	PZ787624	*E. christenseni*	ON245513	96.74	*E. bovis*	KT184372	98.17	*E. arloingi*	ON245514	96.55
GN12	PZ717108	*E. christenseni*	ON245513	98.85	*E. arloingi*	ON245514	98.66	*E. ashata*	KT184373	96.18
GN13	PZ717109	*E. christenseni*	ON245513	98.18	*E. arloingi*	ON245514	98.18	*E. alpacae*	OR576777	98.46
GN14	PZ717110	*E. christenseni*	ON245513	97.51	*E. arloingi*	ON245514	97.32	*E. ashata*	KT184373	97.79
GN15	PZ717111	*E. christenseni*	ON245513	98.85	*E. arloingi*	ON245514	98.28	*E. ashata*	KT184373	96.79
GN16	PZ717112	*E. christenseni*	ON245513	99.23	*E. arloingi*	ON245514	98.85	*E. ashata*	KT184373	96.39
GN17	PZ717113	*E. christenseni*	ON245513	98.78	*E. arloingi*	ON245514	98.78	*Eimeria* spp.	MW528406	98.89
GN18	PZ717114	*E. arloingi*	ON245514	97.7	*E. christenseni*	ON245513	97.51	*E. alpacae*	OR576777	98.28
GN19	PZ717115	*E. christenseni*	ON245513	99.43	*E. arloingi*	ON245514	98.47	*E. ashata*	KT184373	96.59
GN20	PZ717116	*E. christenseni*	ON245513	98.28	*E. arloingi*	ON245514	98.09	*E. bovis*	KT184372	96.39
GN21	PZ717117	*E. arloingi*	ON245514	96.93	*E. christenseni*	ON245513	96.74	*E. alpacae*	OR576777	99.7
GN22	PZ717118	*E. christenseni*	ON245513	98.85	*E. arloingi*	ON245514	98.47	*E. ashata*	KT184373	96.59
GN23	PZ717119	*E. christenseni*	ON245513	98.22	*Eimeria* spp.	MW528406	97.93	*Eimeria* spp.	MW528407	97.63
GN24	PZ717120	*E. arloingi*	ON245514	99.23	*E. christenseni*	ON245513	98.85	*E. ashata*	KT184373	96.59
GN25	PZ717121	*E. arloingi*	ON245514	96.55	*E. christenseni*	ON245513	96.35	*E. alpacae*	OR576777	99.14
GN26	PZ717122	*E. christenseni*	ON245513	99.43	*E. arloingi*	ON245514	98.85	*E. ashata*	KT184373	96.59
GN28	PZ717123	*E. christenseni*	ON245513	99.04	*E. arloingi*	ON245514	98.47	*E. ashata*	KT184373	6.99
GN29	PZ717124	*E. christenseni*	ON245513	99.43	*E. arloingi*	ON245514	98.85	*E. ashata*	KT184373	96.59
GN30	PZ717125	*E. christenseni*	ON245513	99.23	*E. arloingi*	ON245514	98.47	*E. ashata*	KT184373	96.39
GN31	PZ717126	*E. arloingi*	ON245514	96.55	*E. christenseni*	ON245513	98.85	*E. alpacae*	OR576777	99.14
GN32	PZ717127	*E. christenseni*	ON245513	99.62	*E. arloingi*	ON245514	98.85	*E. ashata*	KT184373	96.39
GN33	PZ717128	*E. arloingi*	ON245514	96.44	*E. christenseni*	ON245513	96.35	*E. alpacae*	OR576777	98.4
GN35	PZ717129	*E. christenseni*	ON245513	96.93	*E. arloingi*	ON245514	98.66	*E. ashata*	KT184373	95.18
GN36	PZ768684	*E. christenseni*	ON245513	98.08	*E. arloingi*	ON245514	96.24	*E. alpacae*	OR576777	98.06
GN37	PZ717130	*E. arloingi*	ON245514	9943	*E. christenseni*	ON245513	96.93	*E. ashata*	KT184373	96.59
GN38	PZ717131	*E. christenseni*	ON245513	99.81	*E. arloingi*	ON245514	97.89	*E. ashata*	KT184373	96.18
GN40	PZ717132	*E. arloingi*	ON245514	91.57	*E. hirci*	KX857469	98.85	*E. hirci*	PV435842	95.32
GN41	PZ717133	*E. arloingi*	ON245514	98.47	*E. christenseni*	ON245513	98.47	*E. ashata*	KT184373	96.18
GN42	PZ717134	*E. arloingi*	ON245514	99.62	*E. christenseni*	ON245513	95.55	*Eimeria* spp.	MW528406	99.78
GN43	PZ717135	*E. christenseni*	ON245513	99.23	*E. arloingi*	ON245514	98.08	*E. ashata*	KT184373	96.79
GN44	PZ717136	*E. christenseni*	ON245513	99.62	*E. arloingi*	ON245514	98.66	*E. ashata*	KT184373	96.39
GN46	PZ717137	*E. christenseni*	ON245513	99.23	*E. arloingi*	ON245514	98.66	*E. ashata*	KT184373	96.79
GN47	PZ717138	*E. christenseni*	ON245513	99.84	*E. arloingi*	ON245514	98.47	*E. ashata*	KT184373	96.18
GN48	PZ717139	*E. christenseni*	ON245513	99.84	*E. arloingi*	ON245514	98.47	*E. ashata*	KT184373	96.18

GN49	PZ717140	*E. christenseni*	ON245513	99.62	*E. arloingi*	ON245514	98.66	*E. ashata*	KT184373	96.39
GN50	PZ717141	*E. christenseni*	ON245513	98.09	*E. arloingi*	ON245514	97.9	*E. ashata*	KT184373	97.6
GN52	PZ717143	*E. christenseni*	ON245513	99.81	*E. arloingi*	ON245514	98.47	*E. ashata*	KT184373	96.18
GN53	PZ717144	*E. christenseni*	ON245513	99.04	*E. arloingi*	ON245514	98.85	*E. ashata*	KT184373	96.79
GN55	PZ717145	*E. christenseni*	ON245513	98.47	*E. arloingi*	ON245514	97.9	*E. ashata*	KT184373	95.99
GN56	PZ717146	*E. christenseni*	ON245513	98.47	*E. arloingi*	ON245514	98.28	*E. ashata*	KT184373	96.18
GN57	PZ717147	*E. christenseni*	ON245513	98.47	*E. arloingi*	ON245514	98.28	*E. bovis*	KT184372	96.38
GN59	PZ717148	*E. christenseni*	ON245513	99.81	*E. arloingi*	ON245514	98.47	*E. ashata*	KT184373	96.18
GN60	PZ717149	*E. christenseni*	ON245513	99.23	*E. arloingi*	ON245514	98.66	*E. ashata*	KT184373	96.39
GN61	PZ717150	*E. christenseni*	ON245513	99.04	*E. arloingi*	ON245514	98.66	*E. ashata*	KT184373	96.18
GN62	PZ717151	*E. christenseni*	ON245513	98.85	*E. arloingi*	ON245514	98.85	*E. ashata*	KT184373	96.39
GN63	PZ717152	*E. christenseni*	ON245513	95.4	*E. arloingi*	ON245514	95.21	*E. ashata*	KT184373	94.18
GN64	PZ717153	*E. arloingi*	ON245514	96.74	*E. christenseni*	ON245513	96.55	*E. alpacae*	OR576777	99.35
GN65	PZ717154	*E. christenseni*	ON245513	99.43	*E. arloingi*	ON245514	98.08	*E. ashata*	KT184373	96.59
GN67	PZ717155	*E. christenseni*	ON245513	99.81	*E. arloingi*	ON245514	98.47	*E. ashata*	KT184373	96.18
